# Biodegradable Polymeric Nanoparticles-Based Vaccine Adjuvants for Lymph Nodes Targeting

**DOI:** 10.3390/vaccines4040034

**Published:** 2016-10-12

**Authors:** Alice Gutjahr, Capucine Phelip, Anne-Line Coolen, Claire Monge, Anne-Sophie Boisgard, Stéphane Paul, Bernard Verrier

**Affiliations:** 1 Laboratoire de Biologie Tissulaire et d’Ingénierie Thérapeutique, UMR 5305, Université Lyon 1, CNRS, IBCP, Lyon 69007, France; alice.gutjahr@ibcp.fr (A.G.); capucine.phelip@ibcp.fr (C.P.); anne-line.coolen@ibcp.fr (A.-L.C.); clairemonge.biotech@gmail.com (C.M.); anne-sophie.boisgard@ibcp.fr (A.-S.B.); 2 InvivoGen, Toulouse 31400, France; 3 Groupe Immunité des Muqueuses et Agents Pathogènes, INSERM CIC1408 Vaccinologie, Faculté de Médecine de Saint-Etienne, Saint-Etienne 42270, France; stephane.paul@chu-st-etienne.fr

**Keywords:** vaccine, adjuvant, immunogenicity, polymer, nanoparticles, nanodelivery, lymph node, antigen, molecular adjuvant

## Abstract

Vaccines have successfully eradicated a large number of diseases. However, some infectious diseases (such as HIV, *Chlamydia trachomatis* or *Bacillus anthracis*) keep spreading since there is no vaccine to prevent them. One way to overcome this issue is the development of new adjuvant formulations which are able to induce the appropriate immune response without sacrificing safety. Lymph nodes are the site of lymphocyte priming by antigen-presenting cells and subsequent adaptive immune response, and are a promising target for vaccine formulations. In this review, we describe the properties of different polymer-based (e.g., poly lactic-co-glycolic acid, poly lactic acid …) particulate adjuvants as innovative systems, capable of co-delivering immunopotentiators and antigens. We point out how these nanoparticles enhance the delivery of antigens, and how their physicochemical properties modify their uptake by antigen-presenting cells and their migration into lymph nodes. We describe why polymeric nanoparticles increase the persistence into lymph nodes and promote a mature immune response. We also emphasize how nanodelivery directs the response to a specific antigen and allows the induction of a cytotoxic immune response, essential for the fight against intracellular pathogens or cancer. Finally, we highlight the interest of the association between polymer-based vaccines and immunopotentiators, which can potentiate the effect of the molecule by directing it to the appropriate compartment and reducing its toxicity.

## 1. Why do We Need Adjuvants?

Preventive vaccination is one of the major successes of medicine. It represents one of the most cost-effective health investments and, according to the World Health Organization, saves 2 to 3 million lives every year. However, infectious diseases (such as HIV, *Chlamydia trachomatis*, *Bacillus anthracis* or malaria) remain a leading cause of death worldwide. In the early days of vaccination, heterologous pathogens (nonpathogenic relative of the organism, such as cowpox virus for smallpox vaccination) [1], or attenuated pathogens (with decreased pathogenicity thanks to repeated culturing, such as the tuberculosis vaccine) were used to immunize populations. Those vaccines have a high intrinsic immunogenicity and usually induce asymptomatic infections that generate a life-long immunity similar to the one observed for individuals recovering from a natural infection. However, for many pathogens, this kind of vaccine has not been successfully developed, notably because of safety issues. Inactivated toxins (such as the inactivated tetanus toxin) or inactivated pathogens (such as the inactivated Polio virus), as well as synthetic peptides and recombinant protein subunits are now developed, but they have a poor immunogenicity. Therefore, they require a co-administration with adjuvants in order to elicit a robust immune response. Among various categories of adjuvants, delivery systems enhance antigen uptake by antigen-presenting cells and their migration into lymph nodes (LNs). Indeed, LNs house B and T lymphocytes, and are the site of lymphocyte priming by antigen-presenting cells for the induction of a subsequent adaptive immune response. Delivery systems can increase the persistence into the LNs and promote a mature immune response. There is a need for innovative adjuvants for prophylactic (preventive) vaccination, but also for therapeutic vaccines. Indeed, the use of vaccination to fight infections or cancer has been in the spotlight recently. The induction of a strong CD8^+^ T-cell immunity is central to elicit the destruction of infected or malignant cells [2], and the use of nanoparticulate vaccines seems to induce this kind of immune response through multiple pathways. For these reasons, adjuvantation is a promising strategy to address the challenges for the design of effective vaccines.

## 2. Biodegradable Nanoparticles for Vaccine Delivery

Biodegradable polymeric particles have been extensively studied during the past two decades. Biodegradable polymers are used in various medical applications, such as wound healing, tissue engineering, orthopedic devices, cardiovascular applications or vaccine administration. The use of nanoparticles (NPs) for vaccine administration was termed “nanovaccinology” in 2012 [3] and presents tremendous potential. Additionally, the use of biodegradable polymers for NP production is safe and reliable. They are indeed excellent adjuvants due to their physicochemical properties, which can be tuned to adapt to the desired antigen release profile and immunological response. Particle size can be tuned by changing the polymer concentration and the method of synthesis [4]. For these reasons, biodegradable NPs are ideal vectors for drug and protein delivery, and thus outstanding candidates for the future of vaccine administration [5,6,7,8,9].

### 2.1. Various Polymers for Vaccine Application

Biodegradable polymers are degraded in vivo by enzymatic processes, either hydrolysis or other mechanisms, and the degradation products are further eliminated by the normal metabolic pathways. This simple characteristic means that these materials have a great potential in medicine.

The field of bioengineering offers a wide range of biodegradable polymers to produce NPs. Multiple polymers are already approved by the U. S. Food and Drug Administration (FDA) for some applications, even though no NP formulation has been approved for vaccination so far [10]. Synthetic or natural biodegradable polymers may be used and each family has attractive properties. However, the most popular biodegradable polymers used in vaccine applications are poly lactic-co-glycolic acid (PLGA), poly lactic acid (PLA) and polycaprolactone (PCL), three synthetic polymers.

PLGA is a highly compatible co-polymer of PLA and poly glycolic acid (PGA), and is FDA-approved for diverse applications. It has emerged as an attractive polymer as it offers wide possibilities for sustained drug delivery. For vaccine applications, NPs of PLGA can carry the antigen by encapsulation or surface attachment by covalent or ionic bonding [11].

Together with PLGA, PLA is one of the most widely used polymers for particulate vaccine delivery, as a single polymer [12,13] or as a co-polymer when coupled to polyethylene glycol (PEG) [14] or PLGA [15]. This polymer is FDA-approved and its nanoformulation has already shown its efficacy in stimulating an efficient immune response after parenteral administration [16,17]. One of the major advantages of this anionic polymer is the possibility of encapsulation of hydrophobic molecules. It has been shown that PLA NPs can encapsulate or adsorb on their surface one or several antigens together with immunostimulant molecules like receptor ligands to improve their immunogenic potential [18].

Like PLA and PLGA, PCL is an aliphatic polyester and is of interest for its safety, low cost and compatibility with other polymers [19]. PCL has safe degradation by-products after hydrolysis of its ester linkages. Indeed, unlike PLA and PLGA, its degradation does not lead to the formation of lactic acid, which could affect the bioactivity of the antigen [20]. PCL has often been used for long-term implantable devices due to its slow degradation rate [6].

### 2.2. Polymers and Antigen/Immunopotentiator Association

The physicochemical properties of biodegradable polymers allow several ways to associate NPs and antigens, immunopotentiators or antigens/immunopotentiators together (Table 1). The bioactive molecules can indeed be trapped in the NPs by encapsulation and be released during NP degradation. The molecule of interest can also simply be adsorbed on the surface of the NPs by electrostatic or hydrophobic interactions [21]. This association is easy to perform but only provides a weak interaction with the NPs. This type of association could be interesting if the application requires a rapid release of the immunomodulators. A chemical conjugation provides a slight to strong association with the NPs by either adding a chemical cross-linker such as unmodified or modified polyethylene glycol (PEG), (interesting because of its thiol reactive maleimide), or by direct group association of the antigen with the carboxylic group of the NPs [22]. However, due to the aliphatic nature of these biopolymers, the strategies for conjugation are limited [23], which is why there is no reference to conjugation of an antigen with PCL nanoparticles in the literature. Yet, although no examples of the use of conjugated PCL nanoparticles for immunotherapy were found, conjugation by amide group could be possible. The ease of adsorbing single or multiple antigens or ligands in PLA/PLGA particles (compared with others adjuvants) explains the renewed interest in vaccine approaches using these polymeric nanoparticles [24].

## 3. Influence of Particle Characteristics on APCs Uptake and Targeting to Lymph Nodes

LNs are target organs for vaccine delivery, where B- and T-lymphocytes reside and are activated in the presence of an antigen. To access the LN, antigens can either enter directly or through intermediate antigen-presenting cells (APCs). Two major types of APCs have been described for their ability to naturally uptake and process antigens: dendritic cells (DCs) and macrophages. They have similar functions, but only DCs are able to migrate from tissues to LNs and prime naïve T lymphocytes. In addition to their ability to migrate to the LNs, DCs have capacity to coordinate innate and adaptive immune responses in vivo and are involved in vaccination strategies [25]. Polymeric NPs enhance vaccine accumulation into LNs and promote superior cellular and humoral immunity to a variety of antigens in mice relative to soluble forms of protein and peptide vaccines [26]. Many studies have reported that nanoparticle characteristics such as size, shape or surface properties can significantly influence their biological activity [27,28]. For example, these parameters can affect targeting to specific cells, antigen uptake and the type of immune response induced. In this part, we discuss the influence of those NP properties on APC uptake and LN targeting.

### 3.1. Effect of Nanoparticle Size for APC Uptake and LN Targeting

**Figure 1:**
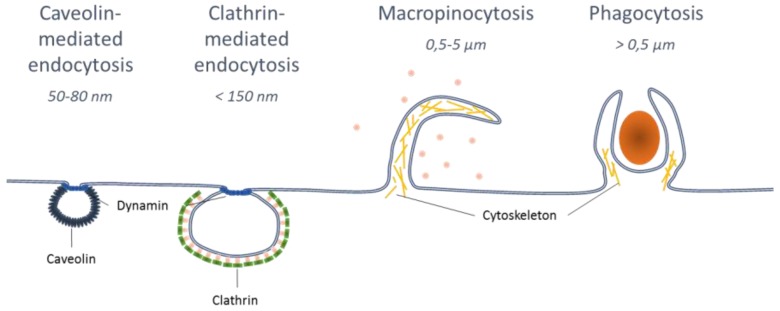
Pathways of endocytosis of exogenous particles, molecules or pathogens. According to the size of an extracellular molecule or particle, different endocytosis pathways take place to engulf it into the cell. Particles between 50 and 80 nm are taken into the cell through caveolin-mediated endocytosis, <150 nm by clathrin-mediated endocytosis, 0.5 to 5 µm through macropinocytosis and larger than 0.5 µm through phagocytosis.

Nanoparticles or exogenous pathogens can be taken up by cells through various pathways. Phagocytosis and pinocytosis (including clathrin-mediated endocytosis, caveolae-mediated endocytosis and macropinocytosis) are the two main endocytic pathways used for NP uptake (Figure 1). These different pathways differ in the composition of the coat, the size of the vesicles and the fate of the internalized molecule [29]. Macropinocytosis is a process for the endocytosis of extracellular material (0.5–5 µm) through membrane protrusions. Phagocytosis is involved in large size endocytic material with size ranges greater than 500 nm. Clathrin-mediated endocytosis induces the uptake of NPs with a size under 150 nm. Caveolae-mediated endocytosis allows different cellular processes including protein endocytosis. Generally, caveolae-vesicles induce the intracellular migration of materials with a size of 50–80 nm. NPs with a similar size as pathogens are efficiently recognized and taken up by APCs for the induction of the immune response [30]. NPs with a size between 20 and 200 nm are preferentially taken up by DCs, through the pinocytosis mechanism, while macrophages uptake larger NPs, from 0.5 to 5 µm, through macropinocytosis and phagocytosis [30].

Although intranodal injections can be performed, they are unusable for prophylactic vaccination, when the vaccine has to be administrated to a large number of persons in a rapid and convenient manner. Thus, alternative ways to direct vaccines to LNs have been developed. NPs can accumulate into LNs directly through lymphatic drainage or be taken up by APCs that will transport them to the LNs. Particle size is one of the most critical factors influencing NP uptake by immune target cells and drainage to the LNs for the induction of an optimal response. Some studies have reported that NPs with a size range between 0.5 and 2 µm are associated with an uptake by DCs at the injection site. Smaller NPs (20–200 nm) are found in LN-resident APCs, suggesting free drainage to the LNs [31]. Another study established that particles larger than 100 nm have difficulty moving into lymphatic vessels and directly transit to LNs [32]. The size limit for lymphatic targeting is not clear and depends on both the tissue site and administration route. However, NPs with a size under 100 nm seem to be an efficient antigen delivery system to target LN-resident DCs and induce adaptive immunity [26].

Particle size can also affect the uptake efficiency and the immune response induction by APCs. Previous studies have established the preferential size of PLGA-based vaccines to induce an effective immune response (Table 2). Smaller PLGA NPs (300 nm) generate a better DC maturation and more efficient Ag-specific immune responses (IgG2a and CD8^+^ T lymphocyte) than 17 µm, 7 µm, 1 µm NPs in vivo in mice [5]. Three hundred and fifty nm PLGA NPs showed an improved internalization over 112 µm microparticles (MPs), correlated with a sustained cellular immune response in a mouse model [33]. Optimal size for efficient uptake and immune induction has not yet been clearly defined. However, nanoparticles seem to be able to trigger either humoral or cytotoxic immune responses depending on their size, probably due to the endocytosis pathway implicated [34,35].

### 3.2. Influence of Particle Shape for Cellular Uptake

Naturally, APCs can internalize various pathogens which can be distinguished by multiple shapes, such as rods, spirals, and ellipsoids. Polymeric delivery systems currently investigated have a spherical shape. However, in recent years, novel methods have allowed the manufacture of polymeric NPs with various shapes [36], and the influence of shape on cellular uptake and biodistribution has been investigated [27,36,37]. Non-spherical NPs show an improved membrane attachment, but a reduced uptake by APCs [17,37]. The non-spherical particle orientation is advantageous for phagocytosis initiation [37], but the membrane wrapping which in theory requires more actin remodeling may explain the more difficult and slower internalization of these particles [17]. Moreover, the shape has an influence on organism biodistribution [38]. This factor is important for the design of NPs for drug delivery at specific sites [39]. This parameter has to be taken into account for cellular uptake and subsequent draining to LNs.

### 3.3. Influence of Surface Characteristics of Polymeric NPs.

Surface properties such as charge and hydrophobicity have been reported to influence the uptake of NPs. Surface charge of NPs plays a significant role in the endocytosis mechanism and the activation of immune responses. Because cell membranes are negatively charged, cationic particles can be more efficiently taken up by APCs than anionic particles. For example, positively charged chitosan-based NPs have an increased internalization rate in eight types of cells, compared to neutral and negatively charged NPs [28]. In all cases, the surface charge of polymeric NPs (cationic or anionic) can be modified by using molecules with various charges like PEG or protamine [40,41]. Although cationic NPs are advantageous to enter into the cells, negative polymeric PLA NPs are also efficiently internalized by DCs [16]. Because positive NPs are more efficiently taken up by APCs, they can migrate through sentinel DCs to LNs, whereas negatively-charged polymeric particles, with a lower cellular uptake rate, might reside at the injection site and can permit antigen or immunopotentiator delivery at the site of injection [28]. Hydrophobicity has also been reported to affect NP uptake and immune response induction. In fact, hydrophobic particles induce a higher immune response than hydrophilic NPs through their increased susceptibility for phagocytosis [7].

In summary, NP size, shape and surface characteristics have an impact on their distribution in the organism, the endocytic processes involved and on the induction of immunity. As modifications in NP properties can induce different responses, the consideration of these parameters is central for adjuvants to target APCs and LNs.

## 4. Immune Responses and Functionalized Nanoparticles

**Figure 2:**
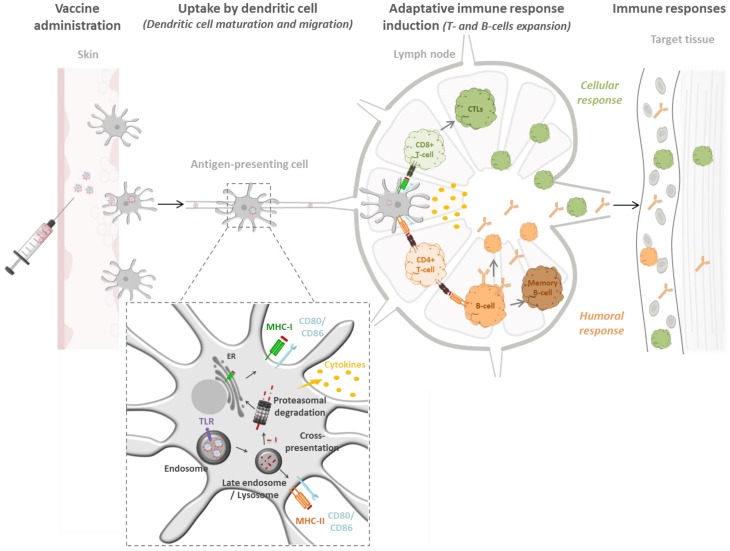
From vaccine administration to immune response. Administered NPs are firstly uptaken by APCs and the antigen is processed to be presented by the appropriate MHC. Antigenic peptide presented through MHC-I induce cytotoxic immune response, the peptides presented through MHC-II can activate CD4^+^ T cells that can provide help for the humoral immune response.

The induction of a protective immunity requires antigen uptake either by circulating APCs present at the vaccine administration site or by LN-resident APCs after antigen migration through lymphatic vessels (Figure 2).

The APC uptake of soluble antigens is often insufficient to induce a protective immunity [42]. As antigen delivery systems, particles have demonstrated a huge potential for the development of vaccines and immunotherapies. The delivery of antigens loaded on NPs has several advantages: it prolongs antigen presence, enhances DC-mediated antigen uptake, directs stimulation of DCs and promotes immune responses [35]. Studies have investigated the advantages of polymeric NPs to induce an immune response in comparison with soluble antigens. For example, the loading of DCs with OVA formulated with PLGA-NPs has been shown to induce a more efficient duplication of CD4^+^ T-cells than soluble OVA (even at 500-times higher dose) [42].

After vaccine administration, antigens coupled to NPs are taken up by APCs (and notably by DCs) directly at the injection site or migrate to LNs through lymphatic vessels to target resident DCs (Figure 2). Via endocytosis, APCs process the foreign materials, induce the presentation of the antigenic fragment on their surface through Major Histocompatibility Complex (MHC) molecules, and activate T-cells responses [43]. Depending on the endocytosis pathways, influenced by NP properties, and on the type of antigen loaded at the surface or into NPs, two types of immune response pathways can be potentially activated (MHC-I or MHC-II).

Studies confirm the added value of NPs as antigen delivery systems to induce broad and potent immune responses. Using an ex vivo priming assay, Petrizzo et al. [42] have demonstrated the efficacy of antigen delivery by PLGA NPs to induce the differentiation of naïve CD4^+^ T cells into memory cells with a TH1 phenotype. Moreover, several antigens can be loaded in the same polymeric NPs. This property allows the induction of a broad immune response. For example, the co-adsorption of HIV p24 antigen and gp120 envelope glycoprotein onto PLA NPs conserves the antigenicity of the proteins and the co-formulation elicits higher antibody titers compared to the gold standard MF59 [44].

Another important goal when designing antigen delivery systems in vaccination is the possibility of obtaining cross-presentation. The cross-presentation process consists of the presentation of an exogenous antigen, normally presented by the MHC-II pathways, in the context of MHC-I [45,46]. Antigens released by NPs in endosomes escape to the cytoplasm, are degraded in molecular fraction by the proteasome and loaded to the MHC-I. The MHC-I/peptide presentation at the APC surface, the expression of CD80/CD86 and the cytokine release induce the differentiation of CD8+ or cytotoxic T-cells [43]. Cross-presentation allows antigen presentation through both routes, and the use of particulate vaccines is one way to facilitate endosomal disruption after internalization [47]. The interest of poly(propylene sulfide) NPs was demonstrated, showing a targeting of LN-resident DCs and the induction of cross-presentation by NPs, leading to a cytotoxic immune response [48]. The delivery of antigens with NPs leads to their presentation through specific intracellular pathways, allowing the control of the type of immune cell stimulated by the APCs and the subsequent immune response [49].

## 5. Nanodelivery of Immunopotentiators

### 5.1. Immunopotentiators as Powerful Vaccine Adjuvants

Two classes of adjuvants are found in most modern vaccines: carrier systems and immunostimulant molecules [50]. As presented earlier, carrier (or delivery) systems, such as polymeric NPs, present the vaccine antigens in an optimal way to the immune system. Immunostimulatory molecules, in contrast to carrier systems, act directly on the immune system to potentiate or orient the immune response against the target antigen.

It is known that the immune system receptors called pathogen-recognition receptors (PRRs) are targeted by conserved microbial products called pathogen-associated molecular patterns (PAMPs). Bacterial cell wall components, viral RNA and CpG DNA are natural pathogen components which activate PRRs. As they are present in most of the whole pathogen-based vaccines, the stimulation of PRRs has been unwittingly used in vaccines for years. Since the early days of the study of innate immunity [51], the interactions between PAMPs and PRRs have been extensively analyzed. Toll like receptors (TLRs), nucleotide-binding oligomerization domain (NOD)-like receptors, RIG-I-like receptors (RLRs) and C-type lectin receptors (CLRs) are the four main groups of PRRs. They sense the presence of infection and activate innate immunity but also have direct effects on the activation of adaptive immunity. Most of the molecular adjuvants currently under evaluation are PRR ligands [52]. The receptors currently targeted by polymeric nanoformulations are mostly endosomal receptors TLR3 [53], TLR4 [54], TLR7/8 [55] and TLR9 [56] (Table 2). TLR3 is stimulated by double-stranded RNA, TLR4 by lipopolysaccharide (LPS), TLR7 and TLR8 by single-stranded RNA and TLR9 by DNA containing CpG motifs (CpG oligodeoxynucleotides, ODN).

Combinations of delivery systems and immunostimulant molecules are commonly being developed as adjuvants because they have the potential to act synergistically to enhance the antigen-specific immune response. Nanodelivery can enhance the immunostimulatory properties of formulated molecules through multiple mechanisms: it can enhance the activity of the adjuvant through its concentration within lymphoid organs, prolonging its exposure to immune cells, it can allow the targeting of the appropriate cellular compartment or change the biological effect of those molecules and protect it from degradation. Moreover, these systems allow the co-delivery of immunopotentiators and antigens, which seems to be valuable for vaccine efficacy as reviewed later.

### 5.2. Improving Nanovectors Efficacy with Molecular Adjuvants

Biodegradable polymer-based nanovectors are often poorly immunogenic and the co-administration with molecular adjuvants is of great interest to improve and/or orient the immune response. For prophylactic vaccination, the co-encapsulation of soluble Leishmania antigens with the TLR4 ligand MPLA (3-O-desacyl-4′-monophosphoryl lipid A), the only TLR ligand approved as an adjuvant in human licensed vaccines preventing human papillomavirus, hepatitis B and malaria infection [57], proved to be an interesting tool for vaccination. The encapsulation of MPLA in 300 nm PLGA nanoparticles enhances the stimulation of DC maturation compared with the formulation alone [58]. The encapsulation of NOD ligands into PLA NPs of approximately 200 nm potentiates their activity. In fact, nanoformulations are more efficient to induce DC maturation in vitro and the co-injection of encapsulated NOD ligands with PLA particles carrying Gag p24 HIV-1 antigen increases antibody response compared to the NPs without molecular adjuvant or co-administered with the free immunomodulator [18]. Similarly, Wischke et al. [59] demonstrated that the encapsulation of NOD agonists into PLGA MPs of approximately 5 µm allows human moDC maturation in vitro, unlike empty MPs or soluble NOD ligand.

Molecular adjuvants have also a great interest for therapeutic vaccination. The association of a poorly immunogenic melanoma antigen with TLR4 agonist in 400 nm PLGA nanoparticles has a potent anti-tumor effect in mice, activating antigen-specific CD8^+^ T cells able to secrete IFN-γ in LNs and the spleen [60]. TLR9 recognizes specific unmethylated CpG motifs present at a high frequency in the bacterial genome but absent from mammalian genomes. The use of PLGA microspheres (1–10 µm) carrying tumor lysates and TLR9 ligand CpG ODN is promising as an anti-tumor vaccine against prostate carcinoma. Indeed, the co-encapsulation of tumor lysates with CpG-ODN induces high cytotoxic responses, leading to a reduction in tumor growth in a mouse model of prostate carcinoma [61]. Biodegradable polymeric NPs are a good alternative to adjuvants based on mineral oils that can be accompanied with adverse effects. In fact, the encapsulation of poly I:C within polyester (poly(D,L lactic-co-hydroxymethyl glycolic acid) nanoparticles of approximately 450 nm with an antigen from HPV-induced malignancies enhances the population of antigen-specific CD8^+^ cells compared to the formulation without poly I:C, with a strong therapeutic effect and no adverse effects [62].

The uptake of the NPs and the entrapped molecular adjuvants can be further enhanced by surface-modifications. Targeting specific DC subsets by functionalizing vaccines with a DC-binding ligand is an innovative approach to enhance LN targeting and vaccine efficacy. The coating of 200 nm PLGA NPs encapsulating TLRs agonists with antibodies recognizing DC-specific receptors enhances the maturation of DCs and the secretion of pro-inflammatory cytokines, as well as the activation of antigen-specific CD8^+^ T cells in vitro, compared to the untargeted formulations. In vivo, a comparable induction of an antigen-specific cytotoxic response is observed with 100-fold lower amounts of adjuvant when the TLR3 and TLR7 agonists are co-encapsulated compared to their administration in soluble form [57]. The surface-modification of 300 nm PLGA nanoparticles with a TNFα mimicking peptide enhances the uptake of the nanoparticles by DCs in vitro [52]. Adjuvants that target DEC-205 or other cell surface molecules expressed by DCs using antibodies coupled to NPs have been shown to enhance accumulation within LNs and immunogenicity in mice [63] and to promote both cellular and humoral immunity in humans [64]. PLGA NPs (200 nm) loaded with TLR3 and TLR7 ligands and decorated with different monoclonal antibodies targeting DEC-205, CD40 or CD11c receptors (expressed on the surface of DCs) were compared. In vitro, the T-cell proliferation of both CD8^+^ and CD4^+^ cells was efficiently induced by DCs loaded with targeted NPs but not by the untargeted ones. Furthermore, vaccination with PLGA NPs targeted to CD40, DEC-205 or CD11c induced efficient in vivo CD8^+^ response, around 80% specific killing, compared to the 40% induced by non-targeted PLGA NPs [65].

The design of adjuvants mimicking pathogens is a good way to induce an immune response. Triggering a combination of TLRs in APCs can have synergistic effects. The simultaneous delivery of MPLA, CpG ODN and the model antigen OVA loaded on 200 nm PLGA particles induces a strong immune response against the model antigen in a mouse model [66]. The localization of the immunopotentiator seems to be a crucial point for vaccine adjuvant design. The stimulation of humoral and antigen-specific T cell responses is enhanced when CpG is encapsulated, compared to its adsorption at the surface of the nanoparticulate vector, showing the interest of mimicking the biology of the pathogens. The stimulation of TLR4 and TLR9 has a synergistic effect when co-delivered in the same particle, especially for cytotoxic response after vaccination in a mouse model [66]. A synergistic increase in the antigen-specific humoral response is observed after the immunization of mice with 300 nm PLGA NPs containing the TLR4 ligand MPL and the TLR7 ligand R837 compared with the molecular adjuvants alone [67].

All these results show that the encapsulation of molecular adjuvants is an effective way to enhance nanoparticulate vaccine immunogenicity.

### 5.3. Delivery of the Immunostimulant Molecules and Antigens to a Target Compartment

The nanodelivery of molecular adjuvants has diverse effects: it can avoid its systemic dissemination, enhance its uptake by APCs and help its transport to the LNs where the DCs can present the antigen to T cells and direct the immune response. The nanoformulation of a TLR9 ligand (CpG ODN) to 30 nm ultrasmall pluronic-stabilized poly(propylene sulfide) (PPS) NPs allows the drainage of the formulation within LNs and the efficient uptake and cross-presentation by DCs in a mouse model. This efficient response induces a strong cytotoxic immune response [68]. In a recent study, Lynn et al. [69] showed that the covalent binding of a TLR7 ligand to a polymer forming NPs in high density induces a high-magnitude and persistent innate immune activation restricted to the LNs, a necessary condition for promoting protective T_H_1 CD4^+^ and CD8^+^ T cell responses, and high antibody titers. The nanoformulation of TLR7 ligands in PLA-PLGA NPs increases cellularity within the draining LNs when the formulation is injected while no cell infiltration is observed when the free ligand is co-administered with the NPs [70]. This property is not limited to polymeric NPs as it has been demonstrated that lipid NPs enhance LN accumulation of stimulator of IFN genes (STING) ligands [71].

Some PRRs, such as TLR3, TLR7 and TLR9 are expressed within the endosomal compartment. The delivery in endosomes of ligands targeting these receptors is possible when using nanoformulations (Figure 2). The entrapment of TLR7 and TLR9 agonists and a model antigen (OVA) within PLGA microsphere (1–30 µm) leads to phagocytosis by APCs and the transfer into endosomal compartments in vitro. The formulations induce strong Ag-specific CD4^+^ and CD8^+^-mediated immune responses in mice after parenteral administration, leading to protective and therapeutic effects [72]. The encapsulation of CpG ODN within poly(γ- glutamic acid)-graf t-l-phenylalanine ethyl ester (γ-PGA-Phe) nanoparticles is a good way to potentiate the effect of CpG ODN. It induces the uptake by macrophages and the internalization of CpG ODN into endo/lysosomes, where TLR9 is expressed. Interestingly, γ-PGA-Phe particles stimulate TLR4, and the co-delivery with a TLR9 ligand synergistically stimulates macrophages in vitro. The formulation induces higher OVA-specific cytotoxic immune response than the co-administration with free CpG ODN in mice [73]. As presented earlier, the size of the particles influences their uptake by DCs. The uptake is essential as it leads to the targeting of TLR by the appropriate agonist into DCs, which induces their maturation. PLGA NPs (350 nm) and MPs (112 µm) carrying a TLR3 ligand (poly I:C) and the model antigen OVA show an improved internalization of NPs over MPs, correlated with a sustained cellular immune response in a mouse model [34].

The delivery of PRR agonists to the right cells (i.e., LN-resident DCs) and to the right compartment (endosomal compartment of the cell, where the appropriate receptor is expressed) is a crucial point for vaccine adjuvant design. The encapsulation of these ligands within particulate vectors is one way to reach this objective.

### 5.4. The Nanodelivery of Immunomodulators Decreases Their Toxicity

Another interesting aspect of using nanoparticulate vectors is the reduction of the amount of immune modulators that can be toxic in high doses, and the reduction of their systemic dissemination after injection. Indeed, soluble molecular adjuvants can induce toxicity due to the rapid clearance to the bloodstream after injection and the induction of a cytokine storm. Their formulation within particles allows local immune responses within the LNs. Synthetic vaccine particles co-encapsulating a model antigen and TLR7/8 or TLR9 ligands co-delivered with the model antigen OVA induce stronger humoral and cellular immune responses with lower systemic production of pro-inflammatory cytokines than the co-injection with a free agonist [70]. The formulation of polymeric NPs with a TLR9 agonist leads to similar effects of the adjuvant with lower doses, thus limiting toxicity in a mouse model [68]. The binding of a TLR7 ligand with polymer particles has recently been studied, the nanoparticulate formulation leading to a reduced systemic innate immunity activation (often associated with adjuvant toxicity and morbidity) and improved vaccine immunogenicity [69]. The encapsulation of TLR3 and TLR7 agonists reduces their side effects and the related toxicity associated with their soluble administration in a mouse model [63].

### 5.5. Co-Delivery or Co-Administration with Antigens?

Different strategies propose to co-deliver within the same particle or to co-administer with two different carriers the antigen and the immune modulator. The co-delivery of a TLR7 agonist with a diphtheria-tetanus-pertussis DTaP antigen results in higher antibody titers when the TLR7 ligand is encapsulated inside PLGA nanoparticles of approximately 350 nm rather than co-administered in a mouse model. The simultaneous delivery of the antigens and the molecular adjuvant is advantageous for the induction of a strong immune response in this model [74]. The co-localization of CpG and antigen on PLGA NPs of around 250 nm in diameter induces much higher antibody titers than the co administration with free CpG motifs in a mouse model. Moreover, it induces a superior protection to West Nile Encephalitis than the control adjuvant alum [75]. The co-delivery of a TLR9 agonist with a model antigen allows a better CD8^+^ immune response than the nanoparticle-conjugated antigen co-administered with the free ligand [68]. The co-delivery of a model antigen OVA and MPLA in 350–450 nm PLGA NPs induces their uptake by DCs, leading to the induction of CD4^+^ and CD8^+^ immune responses in vitro. In mice, this formulation offers clonal expansion of CD4^+^ T cells capable of cytokine secretion [76]. In vivo, the administration of poly I:C or CpG ODN encapsulated with OVA in 1 µm PLGA particles improves the CTL activity of NP-OVA. In a solid tumor model, the functionalized NPs exert potent antitumor activity [77]. The co-entrapment of poly I:C and CpG with OVA in 150 nm mannose-functionalized aliphatic polyester-based nanoparticles induces a long lasting T_H_1 immune response that is not observed with the co-administered free molecules [78]. Interestingly, the release of PRRs agonists and antigens is important for the induction of an appropriate immune response. The comparison of bare, chitosan-coated and protamine-coated PLGA MPs for the coupling with antigen and CpG showed that uncoated MPs are more efficient for the induction of CTL response in mice. The authors assessed that the low release of the antigen and CpG with other formulations was the cause of this impaired activity [79]. To conclude, the co-delivery of immunopotentiator and antigens seems to be a powerful tool to induce a strong immune response.

## 6. Conclusions and Future Perspectives

Polymer-based particulate systems are effective delivery systems, able to deliver immunopotentiators and antigens. They enhance vaccine accumulation into LNs and promote superior humoral immunity to a variety of antigens relative to soluble forms. The nanoformulation allows the process of cross-presentation, leading to the induction of cytotoxic immune response, essential for the fight against intracellular pathogens and cancer. The nanodelivery of immune adjuvants has a great potential for vaccine adjuvantation. It allows the enhancement of the activity of the molecular adjuvant by concentrating it within the LNs, prolonging its exposure to immune cells, while reducing its toxicity. Thus, the co-delivery of antigens and immunopotentiators in polymeric nanoparticles seems to be an efficient way to induce a potent immune response.

A multitude of new challenges for the design of more effective vaccine adjuvants awaits the scientific community, with the manipulation of size, shape and other properties. Local toxicity of polymer-based particulate vaccines could be a detrimental issue, hampering their clinical use, this is why efforts have to be made to overcome this issue. Moreover, the development of delivery systems carrying RNA or DNA has been in the spotlight recently, and the development of polymer-based NPs carrying those molecules would allow their use for RNA and DNA vaccines. Indeed, even if mRNAs present multiple advantages for vaccine formulation, mRNA-based vaccines raise several challenges: protection, stabilization and transport of mRNAs to DCs to induce antigen production. In order to overcome these challenges, the development of polymeric NPs seems to be promising.

## Figures and Tables

**Table 1:** Possibilities to associate antigens or immunopotentiators to nanoparticles. Encapsulation is performed by mixing the bioactive molecule with the polymer during synthesis and leads to very few physical interactions with the nanoparticles (NP). Adsorption of the immunomodulators occurs via electrostatic or hydrophobic interactions and provides a weak association. A stronger association is provided by the chemical conjugation that links the immunomodulator and the NPs via a cross-linker.

Association	Type of Interaction	Polymers Involved
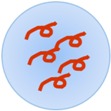 Encapsulation	/	PLA, PLGA, PCL
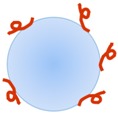 Adsorption	Electrostatic or hydrophobic	PLA, PLGA, PCL
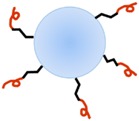 Conjugation	Chemical cross-linking	PLA, PLGA

**Table 2:** In vivo effects of the nanoformulation of molecular adjuvants.

Particles Characteristics (Polymer-Size)	Model (Antigen-Model)	Target Receptor	Co-Administration or Co-Delivery	Immune Response	Ref.
γ-PGA-Phe-200 nm	OVA-mouse	TLR9	Co-delivery	-CD8^+^ T cells response	[73]
HPMA-NIPAM-1 µm	OVA-mouse	TLR7	Co-administration	-CD8^+^ T cells response CD4^+^ T cells response -Antibody	[69]
Mannose-functionalized aliphatic polyester-150 nm	OVA-mouse	TLR3 + TLR9	Co-delivery	-CD8^+^ T cells response	[78]
PLA-200 nm	HIV-1 p24-mouse	NOD1 or NOD2	Co-administration	-Antibody	[18]
PLGA-200 nm	OVA-mouse	TLR3 + TLR7	Co-delivery	-CD8^+^ T cells response -CD4^+^ T cells response -Antibody	[65]
PLGA-200 nm	OVA-mouse	TLR4 + TLR9	Co-delivery	-CD8^+^ T cells response	[66]
PLGA-200 nm	OVA-mouse	TLR3 + TLR7	Co-delivery	-CD8^+^ T cells response	[63]
PLGA-250 nm	rWNVE-mouse	TLR9	Co-delivery	-Antibody -Protection to West Nile Encephalitis	[75]
PLGA-300 nm	OVA-mouse	TLR4 + TLR7	Co-delivery	-Antibody	[67]
PLGA-350 nm	OVA-mouse	TLR3	Co-administration	-CD8^+^ T cells response	[33]
PLGA-350 nm	DTaP-mouse	TLR7	Co-delivery	-Antibody	[74]
PLGA-400 nm	OVA-mouse	TLR4	Co-delivery	-CD8^+^ T cells response -CD4^+^ T cells response	[76]
PLGA-400 nm	Melanoma antigen-mouse	TLR4	Co-delivery	-Anti-tumor effect -CD8^+^ T cells response	[60]
PLGA-1 µm	OVA-mouse	TLR3 or TLR9	Co-delivery	-CD8^+^ T cells response -Antitumor activity	[77]
PLGA-1–10 µm	Tumor lysate-mouse	TLR9	Co-delivery	-CD8^+^ T cells response -Antitumor activity	[61]
PLGA-1–30 µm	OVA-mouse	TLR7 + TLR9	Co-delivery	-CD8^+^ T cells response -CD4^+^ T cells response	[72]
pLHMGA-450 nm	HPV synthetic long peptide-mouse	TLR3	Co-delivery	-CD8^+^ T cells response	[62]
PPS-30 nm	OVA-mouse	TLR9	Co-delivery	-Cross-presentation -CD8^+^ T cells response	[68]

Abbreviations: DTaP, diphtheria-tetanus-pertussis; γ-PGA-Phe, poly(γ-glutamic acid)-graf t-l-phenylalanine ethyl ester; HPMA, *N*-(2-hydroxypropyl)methacrylamide; NIPAM, *N*-isopropylacrylamide; pLHMGA, Poly(d,l-lactic-co-hydroxymethyl glycolic acid); rWNVE, recombinant WN virus envelope protein.
